# The use of traditional and complementary medicine among patients with multiple sclerosis in Belgium

**Published:** 2018

**Authors:** E Huybregts, W Betz, D Devroey

**Affiliations:** * Department of Family Medicine & Chronic Care, Vrije Universiteit Brussel, Belgium

**Keywords:** multiple sclerosis, treatment, traditional and complementary medicine

## Abstract

**Introduction.** Conventional treatment of multiple sclerosis (MS) is often disappointing. As a result, some of these patients seek salvation in traditional and complementary medicine (T&CM). The aim of this study is to describe how many patients with MS use T&CM and what their motives and expectations are in doing so.

**Methods.** Ninety-nine patients with diagnosed MS, attending the service of ambulatory revalidation of the National Clinic for Multiple Sclerosis in Melsbroek (Belgium) were included in February 2004 in this retrospective study. All patients had MS resulting in motoric or psychosocial symptoms. The disability was not quantified for this study. Participants were interviewed by means of a structured questionnaire on their current treatment of MS including T&CM.

**Results.** In total 44% of the participants had experiences with T&CM. The most frequently used T&CM were homeopathy and acupuncture. Participants using conventional treatment were more satisfied with the support (p=0.006) and the treatment outcome (0.018) than T&CM users. The use of T&CM was not related to gender, education, living conditions, causal treatment such as disease modifying-therapy (DMT), grade of disability or subtype of the disease.

**Conclusion.** Patients diagnosed with MS seek hope in T&CM such as homeopathy or acupuncture. The results of this study suggest that MS patients need more professional support in their personal search for alternative therapies.

**Key point.** 50% of patients diagnosed with multiple sclerosis search relief in traditional and complementary medicine such as homeopathy or acupuncture.

These patients often feel compelled to try every opportunity to heal, often stimulated or urged on by friends or relatives.

Multiple sclerosis patients are more satisfied with their conventional treatment than with the traditional and complementary medicine.

## Introduction

Multiple sclerosis (MS) is a chronic disabling neurological disease which often leads to severe disability and many accompanying symptoms. The disease has an unpredictable intermittent course of exacerbation and remission and stress-related conditions can provoke exacerbations [**1**-**3**].

Although much research has been done in the conventional treatment of MS during the past years, the disease still has no definite cure. The present treatments focus on disease-modifying drugs to control the disease process [**4**]. However, the management of the multiple accompanying symptoms remains one of the most important aspects of the treatment.

The intermittent course of the disease with exacerbation and remission and the lack of a definite cure makes the patient vulnerable to traditional and complementary medicine (T&CM). In addition to the dissatisfaction with currently available treatments, the usage of T&CM is also encouraged by anecdotal reports of benefits after T&CM use [**5**][**6**].

Several studies report that MS patients use both T&CM and conventional medicine [**6**-**8**], but no evidence exists on the effects of T&CM on the disease itself [**9**][**10**]. However, the placebo effect, as well as the reduction of stress, might relieve the symptoms of MS exacerbations [**11**][**12**]. The use of T&CM seems to be related to the occurrence of depression and to coping strategies, including the search for information on MS and personal involvement in the treatment process [**13**-**15**].

Despite the subjective effect of T&CM on the quality of life of these patients, physicians should be aware of the side effects of these T&CM. The list of side-effects is very long: some T&CM are very expensive, they can interact with the conventional treatment, they are often provided by non-medically trained people and therefore can result in the interruption of the conventional treatment. Severe cases of organ toxicity are also known to occur [**16**][**17**].

The aim of this study is to describe how patients with MS use T&CM and what their motives and expectations are.

## Methods

*Definition of* T&CM

According to the WHO, T&CM merges the traditional medicine and the complementary medicine and its encompassing products, practices and practitioners [**18**]. For this study within the MS context we used the definition of T&CM as provided by the National Institute of Health: “a group of diverse medical and health care systems, practices, and products that are not presently considered to be part of conventional medicine” [**9**].

***Participants***

Participants for this retrospective study were recruited at the service of ambulatory revalidation of the National Clinic for Multiple Sclerosis in Melsbroek (Belgium) in February 2004. More than 300 MS patients from all regions of Belgium attended the clinic for ambulant treatment and revalidation and only patients with disabling MS attended the clinic. MS was sufficiently severe to impair daily activities and warranting treatment. The disability was not quantified for this study and the disability percentages were determined by the medical officers of the Belgian ministry of health in the context of a disability allowance.

The first one hundred patients who presented themselves were invited to participate in the study and only one patient refused. The 99 remaining participants were interviewed by means of a structured questionnaire and all participants were eventually interviewed because the completion of the written questionnaire was too tiring and demanding for most.

***Questionnaire***

The structured questionnaire inquired into the onset and the course of the disease and the followed treatments since the onset of the disease. Participants who received T&CM were also asked about the initiator of the T&CM, the duration, the influence on the mood, the results and the price. Gender, age, education and living conditions were recorded for all participants.

***Ethical considerations***

The inclusion of patients was done according to the applicable ethical regulations and the permission of the hospital was obtained. The participants were informed about the aim of the study and gave their oral consent and anonymous processing of the data was guaranteed. An approval by an ethical committee was not required for non-interventional and retrospective studies at the time that the study was conducted.

***Statistics***

Analyses were done with IBM SPSS Statistics for Windows (Version 20.0. Armonk, NY: IBM Corp.). For the detection of statistically significant differences between discrete variables the cross-tables and the Chi-Square test were used, while for continuous variables, the T-test was used.

## Results

Demographics

In total 99 participants (58% women) finished the interview and the mean age at the onset of the disease was 32.3 years (SD 10.4). The minimum age at onset was 11.3 and the maximum age was 64.8. Most of the participants lived in a semi-rural area (40%), 35% in a rural area and 25% in an urban area. For most of the participants, secondary school was the highest educational degree (53%), 6% finished only primary school, 29% followed higher education and 12% had a university degree.

***Subtypes of the disease***

The subtype of the disease was known for all participants: 37 were in the relapsing-remitting subtype (RRMS), 26 were in the secondary progressive subtype (SPMS), 36 in the primary progressive subtype (PPMS) and none were in the progressing relapsing subtype (PRSM). Ninety percent of the participants were recognized by the government as disabled, 65% of them had a disability of more than 80%, while the others had a disability of at least 66%.

***Conventional treatment***

In total 46% of the participants were receiving a disease-modifying treatment (DMT) (55% of men and 40% of women; p=0.16). The participants were asked to express their satisfaction with the information on the treatment, support and treatment outcome on a scale from one to ten. Patients who received symptomatic treatment only were more satisfied with the information on the treatment and the treatment outcome than patients who received causal treatment such as DMT **(Table 1)**.

**Table 1:** Satisfaction with explanations of treatment, support and treatment outcome (mean score on max 10 with standard deviation) for (causal or symptomatic) conventional medical treatment of multiple sclerosis

	Causal treatment such as DMT* (n=46)	Only symptomatic treatment (n=53)	p-value
Satisfaction with explanations on the treatment	6.8 (2.6)	7.9 (2.3)	0.028
Satisfaction with support	6.8 (2.6)	7.7 (2.2)	0.065
Satisfaction with treatment outcome	5.8 (3.1)	7.0 (2.7)	0.042

*DMT=Disease Modifying Drugs

***Traditional and complementary medicine***

Forty-four percent of the participants tried at least one T&CM. Among these, 15% tried two T&CM or more and 10% tried at least three T&CM. In a univariate analysis, the use of T&CM was not related to gender, education, living situation, causal treatment such as DMT, disability or subtype of the disease **(Table 2)**.

**Table 2:** The use of traditional and complementary medicine according to gender, age at onset of the disease, education, living situation, causal treatment, the degree of disability and subtype of multiple sclerosis in patients with multiple sclerosis

	Total	T&CM^$^ (n=45)		No T&CM^$^ (n=54)		p-value
	N	n	%	n	%	
Men	42	20	48%	22	52%	0.37
Women	57	25	43%	32	57%	
Age at onset of the disease	32,3	30,6		33,7		0,149
Rural	35	16	46%	19	54%	0.99*
Semi-rural	39	18	46%	21	54%	
Urban	25	11	44%	14	56%	
Causal treatment such as DMT^£^	46	23	50%	23	50%	0.40
No causal treatment	53	22	41%	31	59%	
Primary school	6	2	33%	4	67%	0.96^**^
Secondary school	52	23	44%	29	56%	
Higher education	29	13	45%	16	55%	
University	12	5	42%	7	58%	
Severe disability (>80%)	59	24	40%	35	60%	0.11^*^
Moderate disability (66-80%)	31	14	46%	17	54%	
Mild disability (< 66%)	9	7	78%	2	22%	
Relapsing-remitting subtype (RRMS)	37	19	51%	18	49%	0.66*
Secondary progressive subtype (SPMS)	26	11	42%	15	58%	
Primary progressive subtype (PPMS)	36	15	42%	21	58%	

^*^ p-value for the 3x2 table with 2 degrees of freedom

^**^ p-value for the 4x2 table with 3 degrees of freedom

^$^ T&CM = Traditional and complementary medicine

^£^ DMT=Disease Modifying Drugs

Eleven participants used homeopathy, nine tried acupuncture, four used nutritional supplements, some individuals followed a macrobiotic diet, used bioresonance, drank horse-milk, consumed tolpa peat extract (TPE), used T-cell vaccination (TCV) and had ozone therapy. Seven participants knew they received T&CM, but they could not remember what kind of T&CM and five participants considered a treatment with goat serum. Participants who did not use T&CM did so because their neurologist gave them information on T&CM or because they did not believe in T&CM. Users of T&CM were more satisfied with the support and treatment outcome of their conventional medicine than of their T&CM **(Table 3)**.

**Table 3:** Satisfaction with explanations on the treatment, support and treatment outcome (mean score on max 10 with standard deviation) of the users of traditional and complementary medicine for both conventional and alternative treatment of multiple sclerosis

	Conventional medicine (n=45)	T&CM^*^ (n=45)	p-value
Satisfaction with explanations on the treatment	7.0 (2.8)	6.1 (3.2)	0.16
Satisfaction with support	7.2 (2.4)	5.4 (3.6)	0.006
Satisfaction with treatment outcome	6.5 (2.8)	4.8 (3.7)	0.016

^*^ T&CM = Traditional and complementary medicine

Most of the participants using T&CM were stimulated to do so by friends (29%), family members (18%), a physician (16%) or another MS patient (9%). Newspapers and magazines (4%) and information leaflets (2%) accounted for a minority of the referrals, but 22% of the participants did not remember how they came into contact with T&CM.

In 60% of the participants, T&CM was prescribed by a physician practicing T&CM. In 6% the T&CM was prescribed by a conventional physician and in 34% it was not prescribed by a physician. Two conventional physicians prescribed a non-conventional treatment: it concerns T-cell vaccination, experimental treatment and vitamin supplements, whose effect on the disease is not proven.

Only 45% of the T&CM users indicated that the expectations of the treatment were at least partly fulfilled despite the fact that most T&CM only promised that they would relieve the symptoms. T&CM had a positive influence on the mood of the participants, with seventy-one percent reporting at least a temporarily improved mood.

## Discussion

***The use of* T&CM *in MS***

To this day, MS is an incurable neurological disabling condition and physicians have the obligation to communicate this to the patients. Accepting incurable illness may be associated with a loss of hope, and consequently not only the patient but also his environment - friends, relatives and physicians - are constantly searching for a cure.

Forty-four percent of the participants have tried T&CM. This is remarkably lower than in several other studies where the proportions of MS patients using T&CM reached up to 77% [**14**][**19**]. Results similar to our study were found in Sweden where 46% of MS patients tried T&CM [**20**]. However, some studies also report a lower use of T&CM, for example, 37% among US-veterans [**21**].

Our study population probably included more severely affected MS patients, as compared to other studies. This may be related to the fact that all patients were recruited at the MS Clinic where only severely affected MS patient attend. In the study of Apel et al., users of T&CM were more severely affected by the MS than non-users [**22**]. This confirms, at least partly, that the lower rate of T&CM use that was found in our study is not related to the severity of the disease among our participants.

There was no statistical association between the use of T&CM and education, place of residence, time of onset of the disease, the degree of disability or subtype of the disease. In contrast to our findings, MS patients in Denmark were more likely to use T&CM if they were female, of a younger age, educated at bachelor level or above, and had a high income [**23**].

***Referral to* T&CM**

Most users of T&CM did so on the advice of their family and friends. The prescriber was in most cases a physician who was familiar with T&CM and it is also noticeable that a significant proportion of MS patients treated themselves with T&CM. From other studies, it is known that the major reason for choosing T&CM is the fact that the conventional treatment was not effective. Also, anecdotal reports of other T&CM users and doctor referrals led to other patients using T&CM [**1**]. T&CM users communicated very little with their physician about the T&CM treatments used [**23**][**24**] and they were afraid to do so because they feared a negative judgment by their physician. This lack of communication between the patient and the physicians and other healthcare professionals may hamper the conventional therapies and subsequently the outcome.

***Types of* T&CM**

The list of T&CM used to treat MS is almost inexhaustible. Most T&CM users in our study used homeopathy and acupuncture, but also osteopathic manipulations, vitamins/minerals, nonvitamin-nonmineral-natural products, special diets, reflexology, aromatherapy, omega-3 fatty acids, removal of amalgam fillings and selenium were used [**14**,**19**,**25**-**27**]. Some also tried new and more specific treatments such as tolpa peat extract and bioresonance.

The use of massage, yoga, relaxation and meditation was also often used among MS patients. As these were mainly used as a way to cope with the many symptoms of the disease such as fatigue, weakness and involuntary muscle spasms, there were arguments to classify these kinds of treatments under well-being treatments rather than under T&CM [**26**].

***Evidence for* T&CM *in MS***

Several small studies with questionable methodology claim that some T&CM might have an effect on the evolution of the disease or at least that they improve the symptoms [**26**][**28**][**29**].

The 2014 report of the guideline development subcommittee of the American Academy of Neurology reported on several types of T&CM in the treatment of MS [**30**]. They advised that magnetic therapy is probably effective for fatigue but probably not effective for depression. Fish oil is probably not effective in MS for relapses, disability, fatigue, MRI lesions, and quality of life (QOL). Ginkgo biloba is not effective in MS patients with cognitive impairment, but it is possibly effective on fatigue symptoms in MS patients. Furthermore, the report concluded that reflexology is possibly effective on paraesthesia caused by MS.

Cari Loder, an MS patient herself, reported in the mid-nineties that the combination of her antidepressant lofepramine, vitamin B12 and the amino acid phenylalanine relieved her MS symptoms. However, the report concluded that her regimen is possibly ineffective for disability, symptoms, depression, and fatigue. Thereupon bee sting therapy is possibly ineffective in MS patients for relapses, disability, fatigue, lesion burden/volume, and health-related QOL. Another report concluded that reflexology was not an effective treatment for MS or any other medical condition [**24**].

Although it is generally assumed that T&CM practitioners offer better information and support than the conventional physicians, this is not reflected in our study. On the contrary, in our study participants using the conventional treatment were more satisfied with the support and the treatment outcome than T&CM users. The higher satisfaction among users of the conventional treatment was not related to differences related to gender, the age of onset of the disease, education, living situation, causal treatment, the degree of disability and subtype of multiple sclerosis (**Table 2**).

The MS patient usually has no idea whether or not the advised treatment is evidence-based. An example is a participant that received T-cell vaccination, a treatment which was in an experimental stage.

The U.S. Food and Drug Administration recently removed acupuncture needles from the category of experimental medical devices and now regulates them just as it does for other devices, such as surgical scalpels and hypodermic syringes, under good manufacturing practices and single-use standards of sterility. Although the devices are regulated, this does not represent evidence on the treatment with acupuncture. In this paper, we still consider acupuncture as a T&CM in accordance with the World Health Organization who classifies acupuncture as traditional and/or complementary [**18**].

***Limitations of the study***

Although the questionnaire was designed to be completed by the participants themselves, the pilot study showed that it was, for most participants, too demanding to personally fill in. For that reason, the questionnaire was completed by one of the investigators (EH) during the interviews with the participant.

For some patients, it was also difficult to remember what kind of T&CM they received or to make the difference between T&CM and conventional therapy.

Since the start of our study, the treatment strategy for MS has changed in many aspects. The conventional therapies with anti-inflammatory and immunomodulatory therapy were the preferred treatment for many decades. Promising new therapies with stem cell transplantation and other new therapeutic strategies have been proposed [**31**]. However, there is still a need for effective therapies for progressive multiple sclerosis. For many patients, the worsening of the disease cannot be prevented, nor can the damage be repaired or the function loss to be restored [**32**]. It is therefore likely that many MS patients still seek relief in T&CM.

## Conclusions

Almost one in two patients diagnosed with an incurable disease such as MS search relief in T&CM and the two main methods are through homeopathy or acupuncture. No T&CM has ever proved any positive influence that is more than placebo on the course or symptoms of the disease.

MS patients are more satisfied with their conventional treatment than with the T&CM. However, MS patients often feel compelled to try every opportunity to heal, often stimulated or urged on by friends or relatives.

T&CM therapists are very modest in promising results to MS patients and furthermore, the promised results are achieved only in a minority of cases.

This study shows that the MS patient needs reliable information and support and that the healthcare providers, not only the neurologist but especially the family physician, with their closer patient/doctor relationship, is ideally placed to provide this support.

**Acknowledgments**

The authors thank the National Multiple Sclerosis Center in Melsbroek for their collaboration, Guy Sermeus from the consumers’ organisation Test-Aankoop for the data analysis in SPSS, Chris van Hoeymissen and Harry Audenaerd for the layout of the tables, Dr Veronique Bisset for the scientific advice and David Proot and Babs Sybesma for the English language editing.

## Conflict of Interest

The authors declare that there is no conflict of interest.

## Appendix 1: Questionnaire (translated from Dutch)

**Section 1: Questions about the course of the disease**

**In which year did you first experience symptoms of MS?**

**Are you recognized by the Ministry of Social Security as disabled?** ○ yes ○ no

If so, what percentage? ○ 80% ○ 66% ○ 50% ○ less than 50%

**How does your disease progress?** ○ outages and periods of recovery ○ deterioration without periods of recovery ○ first with a periodical recovery, but now only a decline ○ I did one outage with practically complete recovery ○ I am stable: since ... (number of years) and/or ... (number of months)

**Section 2: Experience with the treatment of MS:**

**Indicate which treatment you have already followed:** ○ No treatment

A conventional treatment with: ○ Interferon ○ A.C.T.H. ○ Copaxone (=glatiramer acetate) ○ Imuran (=azathioprine) ○ Mitoxantrone ○ Endoxan (=cytophosphane)

A traditional or complementary treatment (arranged alphabetically): ○ Acupuncture ○ Bee sting ○ Chiropractic treatment ○ Diet: Which? ............................................. ○ Dietary supplements ○ Evening primrose oil ○ Evers diet ○ Herbal treatment ○ Homeopathy ○ Horse milk ○ Kousmine diet ○ Nieper diet ○ Paranormal healer ○ Pilgrimage ○ Praying ○ Prokarin (=histamine and caffeine) ○ Tea ○ Tolpa ○ Vitamin supplements

Another treatment: Which? (fill in) 1). . . . . . . . . . . . . . . . . . . 2). . . . . . . . . . . . . . . . . . . 3). . . . . . . . . . . . . . . . . . .

Are you considering another treatment? ○ No ○ Yes: . . . . . . . . . . . . . . . . . . . . . . . . . . . . . . . . . .

**Questions concerning your experience with the conventional treatment of MS by your physician (neurologist)**

(Give a rating from 0 (very dissatisfied) to 10 (very satisfied)).

Are you satisfied with the explanation you received?

0 – 1 – 2 – 3 – 4 – 5 – 6 – 7 – 8 – 9 – 10

Are you satisfied with the guidance you received?

0 – 1 – 2 – 3 – 4 – 5 – 6 – 7 – 8 – 9 – 10

Are you satisfied with the result of the treatment?

0 – 1 – 2 – 3 – 4 – 5 – 6 – 7 – 8 – 9 – 10

**Section 3: Experience with traditional or complementary treatments of MS**

If you did not follow a complementary treatment -> go to section 4

**Which traditional or complementary treatment was the most important to you?**

. . . . . . . . . . . . . . . . . . . . . . .

**How many years after the diagnosis have you started this treatment?** ○ in the first 5 years ○ after the first 5 years

**At what stage of your disease was that?** ○ During a crisis: ○ During a stable phase ○ During a period of decline:

**Questions concerning your experience with the traditional or complementary treatment of MS by your physician (neurologist)** (Give a rating from 0 (very dissatisfied) to 10 (very satisfied)).

Are you satisfied with the explanation you received?

0 – 1 – 2 – 3 – 4 – 5 – 6 – 7 – 8 – 9 – 10

Are you satisfied with the guidance you received?

0 – 1 – 2 – 3 – 4 – 5 – 6 – 7 – 8 – 9 – 10

Are you satisfied with the result of the treatment?

0 – 1 – 2 – 3 – 4 – 5 – 6 – 7 – 8 – 9 – 10

**Who advised you this treatment?** ○ a friend or acquaintance: ○ a family member ○ a doctor (general practitioner or specialist), which:. . . . . . ○ an article in newspaper or magazine, which:. . . . . . ○ colleagues ○ another person with MS ○ the MS patient association ○ the alternative healer himself ○ a folder ○ a message on the internet ○ in another way, which. . . . . . . . . . . . . . . .

**Who prescribed the treatment?** ○ a doctor who mainly uses conventional treatments ○ a doctor who mainly uses complementary treatments ○ a pharmacist ○ a physiotherapist ○ someone else: . . . . . . . . . . . . . . . . . ○ nobody

**How long did you follow the treatment?** ○ less than a month ○ less than a year ○ more than a year ○ I have been working on the treatment since: . . . . . . . . . . . .

**What result was promised?** ○ full cure ○ a chance of complete cure ○ partial improvement: ○ a chance of partial cure ○ no further deterioration ○ another result: . . . . . . . . . . . .

**Was the promised result achieved? (circle your answer)** ○ completely ○ partially ○ not at all

**Did you experience any side effects of the treatment?** ○ no ○ yes, but they did not bother me ○ yes, they were annoying, but I continued the treatment ○ yes, they were the reason I stopped

**What was the most annoying side effect?** ○ pain ○ nausea ○ the treatment was too difficult ○ another side effect: . . . . . . . . . . . .

**Has the treatment improved your mood?** ○ yes, permanently (also after the treatment) ○ yes, temporarily (during the treatment) ○ no, my mood did not change ○ no, my mood deteriorated

**Would you recommend the treatment?** ○ yes ○ no

**How much did the treatment cost?** ○ less than 25 € ○ between 25 and 250 € ○ between 250 and 2500 € ○ between 2500 and 125000 € ○ more than 125000 €

**Section 4: Personal information**

**In which year were you born?** 19..

**Which is your gender?** ○ male ○ female

**In what type of area do you live?** ○ rural ○ semi-rural ○ urban

**What is the highest level of education that you have completed?** ○ primary education ○ secondary education up to the 1st degree ○ secondary education up to the 2nd degree ○ secondary education up to 3rd degree ○ higher non-university education ○ higher university education
